# Role of Matricellular CCN Proteins in Skeletal Muscle: Focus on CCN2/CTGF and Its Regulation by Vasoactive Peptides

**DOI:** 10.3390/ijms22105234

**Published:** 2021-05-15

**Authors:** Daniela L. Rebolledo, María José Acuña, Enrique Brandan

**Affiliations:** 1 Centro de Envejecimiento y Regeneración, CARE Chile UC, Pontificia Universidad Católica de Chile, Santiago 8331150, Chile; mjose.acuna@ubo.cl; 2 Centro de Excelencia en Biomedicina de Magallanes (CEBIMA), Universidad de Magallanes, Punta Arenas 6213515, Chile; 3 Centro Integrativo de Biología y Química Aplicada (CIBQA), Universidad Bernardo O Higgins, Santiago 8370854, Chile; 4 Departamento de Biología Celular y Molecular, Facultad de Ciencias Biológicas, Pontificia Universidad Católica de Chile, Santiago 8331150, Chile; 5 Fundación Ciencia & Vida, Santiago 7810000, Chile

**Keywords:** cellular communication network, CCN, skeletal muscle, vasoactive peptides, fibrosis, CCN2/CTGF, KKS

## Abstract

The Cellular Communication Network (CCN) family of matricellular proteins comprises six proteins that share conserved structural features and play numerous biological roles. These proteins can interact with several receptors or soluble proteins, regulating cell signaling pathways in various tissues under physiological and pathological conditions. In the skeletal muscle of mammals, most of the six CCN family members are expressed during embryonic development or in adulthood. Their roles during the adult stage are related to the regulation of muscle mass and regeneration, maintaining vascularization, and the modulation of skeletal muscle fibrosis. This work reviews the CCNs proteins’ role in skeletal muscle physiology and disease, focusing on skeletal muscle fibrosis and its regulation by Connective Tissue Growth factor (CCN2/CTGF). Furthermore, we review evidence on the modulation of fibrosis and CCN2/CTGF by the renin-angiotensin system and the kallikrein-kinin system of vasoactive peptides.

## 1. The CCN Family of Matricellular Proteins

The CCN family of matricellular proteins is a group of six proteins that share conserved structural features and have diverse biological roles [1,2]. This family of proteins was initially named by the first letter of the three first discovered members of the family: cysteine-rich protein 61 (CYR61), connective tissue growth factor (CTGF), and neuroblastoma overexpressed (NOV). Later, three more members were described: the Wnt-inducible signaling proteins WISP-1, WISP-2, and WISP-3. After years of discussion on the nomenclature, an agreement was reached to unify the family’s name and proteins members to avoid misunderstandings given the several different denominations found in the literature. This protein family was renamed CCN, the acronym for cellular communication network factors (CCNs). Its six members were named in the order they were discovered: CCN1 (CYR61), CCN2 (CTGF), CCN3 (NOV), CCN4 (WISP-1), CCN5 (WISP-2), CCN6 (WISP-3) [3,4].

CCNs are structurally characterized by four very well conserved modules (I to IV). Module I, close to the N-terminal, corresponds to the insulin-like growth factor binding protein-like (IGFBP), which is followed by module II, the Von Willebrand factor type C repeat (VWC). Next, there is an unstructured “hinge” region of variable length, which is susceptible to cleavage by various proteases. The hinge region connects with the III domain, the thrombospondin 1 type I repeat (TSP1). Last, module IV, the carboxy-terminal cysteine knot (CT), is missing in CCN5/WISP-2. Different variants of each CCN member have been found at the protein and mRNA level, product of protein cleavage or alternative splicing [3,5,6,7].

Although some CCNs were recognized first as growth factors, today it is understood that they function more as regulators of several signaling pathways. Regarding this regulatory role, CCN proteins have no described canonical receptors but can interact with numerous transmembrane proteins such as integrins, lipoprotein-related receptors, and tyrosine kinase receptors. They can also interact with heparan and sulfate proteoglycans and with extracellular matrix components such as decorin and fibronectin, soluble growth factors, and other matricellular proteins in the intercellular space. Furthermore, the interaction with these proteins modulates their availability and the signaling pathways in which they are involved, in a manner highly dependent on the cell type and the environmental context in which they occur [3,6,8,9]. Consequently, CCNs are currently known to participate in different tissues and organs, with roles in cell adhesion, proliferation, differentiation, migration, and survival [3,5,6].

From their role in normal physiology, alterations in CCNs levels and activity have shown to be linked to several pathologies including, but not limited to cancer [10,11], chronic inflammatory diseases [12], autoimmune disorders [13,14], reproductive diseases [15], metabolic disorders [16], fibrosis in different organs [17,18,19,20], neurodegenerative and neuromuscular diseases [21,22,23]. CCN1/CYR61 is a widely recognized inductor of angiogenesis, improving blood perfusion and oxygen availability, which has been extensively investigated in some tissues such as the retina and the cardiovascular system [24,25]. CCN2/CTGF is highly expressed during embryonic development, where it is involved in several processes, being especially important in the formation of bone and cartilage [26]. Postnatally, CCN2/CTGF levels are low, but they increase during damage or chronic disease in different tissues and organs, playing a role in modulating the extracellular matrix and the fibrotic response [8,27,28,29,30]. CCN3/NOV is also known for promoting angiogenesis by supporting endothelial cell migration, adhesion, and survival [31]. CCN4/WISP-1 and CCN5/WISP-2 have roles in metabolic disorders involving glucose homeostasis, such as insulin resistance, diabetes, and obesity [16,32,33], and have been associated with the development and regulation of different types of cancers [34,35,36]. CCN6/WISP-3, on the other hand, has been shown to work as a tumor suppressor blocking breast cancer development [37,38,39].

Although most of the CCN family members are expressed in skeletal muscle during development or adulthood in mammals, not all of them have been deeply studied. This work reviews what is known about CCN proteins’ function in skeletal muscle physiology and disease. Furthermore, we focus on the role of CCNs, especially CCN2/CTGF, in fibrosis and its modulation by vasoactive peptides.

## 2. CCN Proteins in Skeletal Muscle Function and Disease

**Figure 1:**
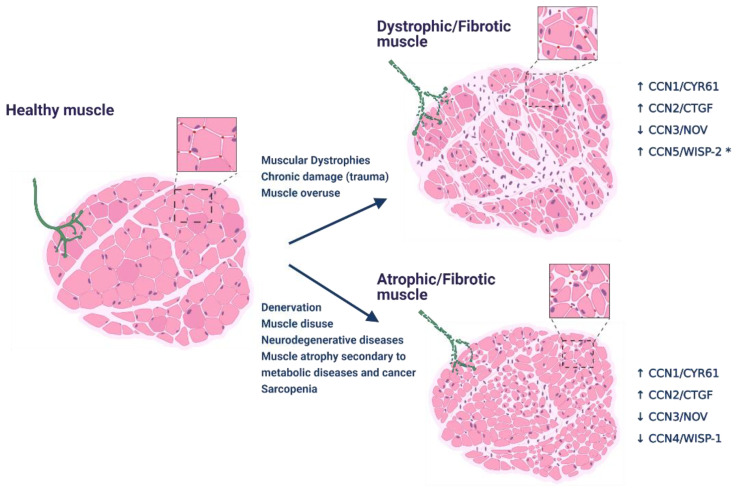
Pathological changes associated with neuromuscular diseases are concomitant with alterations in the levels of some CCNs. The healthy skeletal muscle presents myofibers of homogenous size with peripheric nuclei, accompanied by communication with motor neurons through normal neuromuscular junctions (NMJ). Muscle fibers are surrounded by regular levels of ECM, and few interstitial cells. Surrounding each fiber, capillaries assure the availability of nutrients and oxygen (inset, red dots). We classified different muscle pathologies into two groups schematized on the right. The picture on top shows significant heterogeneity in the size of myofibers and centrally located nuclei due to consecutive rounds of degeneration/regeneration. There is an increased number of interstitial cells, corresponding to cells from the immune system and myofibroblasts that contribute to the excessive accumulation of ECM proteins. Capillary density is lower, which limits oxygen availability. Generally, the NMJ is also compromised. These characteristics are present in muscular dystrophies, muscle overuse, and chronic muscle damage. At the bottom, the whole muscle and myofibers are smaller, and motor neurons and NMJ are significantly affected. There is an increment in the number of interstitial cells, significantly increased ECM accumulation, and reduced capillary density. These features are frequent in muscle atrophy due to neurodegenerative diseases, denervation, disuse, metabolic diseases, cachexia, and sarcopenia. CCNs alterations in these two groups of skeletal muscle diseases are indicated. Up and down arrows indicate up- and down regulation, respectively, at mRNA and/or protein levels (* extrapolated from cardiac muscle disease). Figure created with BioRender.com (accessed on 14 May 2021).

**Figure 2:**
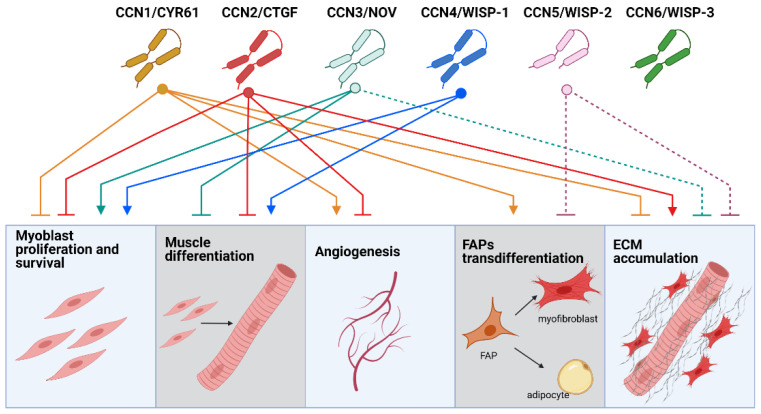
Schematic representation of the described roles for CCNs in skeletal muscle function. Each CCN connects with an arrow when it facilitates a specific process and connects with a blunt line when it inhibits a process. Dotted lines correspond to extrapolated data from cardiac muscle, which has not yet been confirmed in skeletal muscle. Figure created with BioRender.com (accessed on 14 May 2021).

In smooth muscle cells and fibroblasts, some CCNs can be modulated by mechanical stimulation [40,41], which might also be the case in the skeletal muscle. In humans, regular exercise leads to transient upregulation of mRNAS for *Ccn1/Cyr61* and *Ccn2/Ctgf* in skeletal muscle vastus lateralis [42]. CCN1/CYR61 and CCN2/CTGF mRNAs and proteins are also increased in the vastus lateralis muscle, after a single boost of strenuous jumping exercise with high mechanical load [43]. The increased expression in response to physical activity can be linked to a role in the adaptative response to exercise. Nevertheless, pathological conditions affecting skeletal muscle, such as muscular dystrophies and muscle atrophy of different etiology, are also accompanied by alterations in some CCNs (Figure 1). This evidence, together with gain- and loss-of-function strategies, are unveiling the role of CCNs in several aspects of muscle physiology and disease, which we explore below and summarize in Figure 2.

### 2.1. CCNs in Skeletal Muscle Regeneration

The CCN family has been shown to have roles in myoblast proliferation, differentiation, motility, and adhesion, all necessary processes for normal muscle development and regeneration. In vitro studies of muscle progenitor cells (MPCs) treated with CCN1/CYR61 show decrease proliferation [44] and motility [45] of these cell type, which can affect satellite cell quantity and migration capacity during muscle regeneration. CCN3/NOV is expressed during development in skeletal muscle [46]. Its role in the development of this tissue is evidenced by the skeletal muscle atrophy, among other features, caused by the deletion of the *Ccn3* gene [47]. In vitro studies in C2C12 myoblasts have shown that CCN3/NOV promotes myoblast adhesion by increasing FAK phosphorylation. Furthermore, it promotes myoblast proliferation and survival when combined with other growth factors such as FGF2 and IGF-1 [48]. On the other hand, overexpression of CCN3/NOV inhibits differentiation of C2C12 and human primary myoblasts [49]. Together, these results suggest a role for CCN3/NOV in defining cell fate, perhaps by maintaining the satellite cell pool during skeletal muscle formation and also probably during regeneration.

C2C12 myoblasts and myotubes express CCN2/CTGF when exposed to TGF-β and lysophosphatidic acid, and respond to exogenous CCN2/CTGF [50,51,52]. Besides induction of different ECM proteins, CCN2/CTGF reduces early and late differentiation markers. Furthermore, CCN2/CTGF induces dedifferentiation of committed myoblasts, evidenced by the downregulation of MyoD and desmin [52], and the induction of the myofibroblast marker alpha-SMA [51]. More recently, it was proposed that CCN2/CTGF promotes early differentiation but inhibits late differentiation of C2C12 myotubes [53]. The same work explored the myogenic potential of myoblasts obtained from E18 *Ccn2* KO mice. The authors described that proliferation and differentiation of primary *Ccn2* KO myoblasts are impaired compared to wild-type myoblasts and suggest that this situation may also occur in vivo [53]. However, further investigation on *Ccn2* KO mice is not possible postnatally, as they die soon after birth due to severe skeletal malformations that are incompatible with respiration [54]. In our studies using *Ccn2* hemizygous mice (*Ccn2/Ctgf^+/−^*), which have apparently normal skeletal development, we have not found differences in skeletal muscle compared to wild-type under normal conditions. The changes appeared as a consequence of a challenge. In this case, decreased CCN2/CTGF is beneficial for the skeletal muscle, improving muscle regeneration by way of reducing fibrosis [55], which we will discuss later.

Some evidence demonstrated a role for CCN4/WISP in maintaining myogenic potential and promoting skeletal muscle regeneration. For example, CCN4/WISP1 promotes skeletal muscle cell differentiation in bovines by inhibiting TGF-β signaling [56]. Interestingly, Lukjanenko et al. showed that CCN4/WISP-1 is secreted by fibroadipogenic progenitors (FAPs). FAPs are interstitial mesenchymal cells, with critical roles in skeletal muscle physiology and disease, with the potential to differentiate into ECM-producing myofibroblasts or adipocytes [57]. CCN4/WISP-1 secreted by FAPs acts on muscle progenitors promoting their amplification and differentiation through the activation of Akt signaling. During the aging process, CCN4/WISP secretion from FAPs is reduced, contributing to impaired muscle regeneration in the elderly, one of the causes of decreased muscle mass or sarcopenia. The authors show that restoring CCN4/WISP1 levels restores the myogenic potential in aged mice [58].

### 2.2. CCNs Role in Skeletal Muscle Atrophy

Skeletal muscle atrophy in various species has been related to CCN1/CYR61 upregulation: CCN1/CYR61 mRNA, protein, or both, are increased in atrophic mouse models of type-I diabetes [59], atrophy due to malnutrition in bovines [60], muscle waste in patients with chronic obstructive pulmonary disease (COPD) [61], atrophic muscle in mouse model of Amyotrophic lateral sclerosis (ALS) [62], and aging-related sarcopenia in mice and rats [44]. CCN1/CYR61 expression is also increased in the serum of chronic kidney disease (CKD) patients, which present sarcopenic obesity, and in serum and skeletal muscle from a mouse model of CKD [63]. Although more studies are needed to understand if the upregulation of CCN1/CYR61 is a cause or a consequence of muscle waste, some studies suggest that its role in muscle atrophy associates with its possible involvement in decreasing skeletal muscle regeneration, as mentioned before [44,45]. Furthermore, treatment of MPCs with CCN1/CYR61 induces the expression of senescence-associated β-galactosidase, a known marker for cellular senescence that can be linked to CCN1/CYR61 role in aging-related sarcopenia [44]. Treatment of skeletal muscle FAPs with CCN1/CYR61 leads to differentiation into adipocytes, which is linked to sarcopenic obesity in the CKD patients mentioned before [63]. Furthermore, transforming growth factor-β (TGF-β), which is abundant in damaged skeletal muscle, induces FAPs to increase CCN2/CTGF levels together with several markers of transdifferentiation into myofibroblasts [64]. As mentioned earlier, FAPs are a source of CCN4/WISP-1, which promotes skeletal muscle regeneration [58]. Transdifferentiation into adipocytes or myofibroblasts can reduce FAPs population, decreasing normal CCN4/WISP secretion, and contributing to reduced muscle mass. CCN2/CTGF has also been found elevated in models of aging-related sarcopenia [44,65]. However, further investigation is needed to determine whether CCN2/CTGF can directly modulate muscle mass, or if its role in sarcopenia is related to the regulation of muscle differentiation (described above) or muscle fibrosis (discussed later). While no much more is known about other CCNs in skeletal muscle atrophy, CCN5/ WISP-2 was suggested to have a role in the regulation of muscle mass because it was found to be increased by testosterone in a study on the regulation of muscle mass by endogenous and synthetic anabolics [66]. However, there has been no further research on the topic.

### 2.3. CCNs in Angiogenesis and Blood Perfusion of Skeletal Muscle

The microvascular network around tissues, including the skeletal muscle, is essential to deliver nutrients and oxygen for normal function and regeneration. Insufficient oxygen supply has been linked to damage or disease in skeletal muscle [67,68]. Chronic hypoxia might compromise muscle function and has been associated with muscle atrophy and reduced force [69]. In this context, some CCN family members have roles in maintaining the capillary network and blood perfusion. 

CCN1/CYR61 and CCN2/CTGF mRNA and protein levels are increased in vastus lateralis after a single boost of strenuous jumping exercise. This increased expression is concomitant with the increment of angiogenic factors such as vascular endothelial growth factor (VEGF) proteins and hypoxia-inducible factor 1-α (HIF-1α), an angiogenic response to exercise training, which is part of the muscle adaptation to exercise [43]. Consistent with the proangiogenic role of CCN1/CYR61 described in several tissues [24,25], its expression through adenoviral infection leads to improved skeletal muscle angiogenesis and blood perfusion, even better than VEGF expression, in a hindlimb rabbit model of ischemia [70]. More recently, the proangiogenic role of CCN1/CYR61 in skeletal muscle was evaluated using a patch containing exogenous CCN1/CYR61 in a rabbit model of trauma with associated ischemia and increased compartmental pressure. The local administration of CCN1/CYR61 led to improved muscle force and increased angiogenesis after the trauma, with a moderate reduction in fibrosis [71].

Increased CCN1/CYR61 in serum and skeletal muscle in some chronic diseases might be a compensatory response to increase blood supply to the skeletal muscle and other organs. Interestingly, this is usually accompanied by the upregulation of CCN2/CTGF. For example, CCN1/CYR61 and CCN2/CTGF are basally increased in a mouse model for type-I diabetes, which presents myofiber atrophy and reduced capillary number to fiber ratio, evidencing decreased blood supply to the skeletal muscle. However, in this diabetes model, exercise training does not upregulate CCN1/CYR61 nor does restore capillarization in type-I diabetic mice [59,72]. The increased expression of CCN2/CTGF, primarily associated with chronic pathologies, is one factor to be considered concerning this effect. We have shown that hypoxia synergizes with TGF-β to upregulate CCN2/CTGF in skeletal muscle, especially in myofibers [67,68]. While our focus was the effect on skeletal muscle fibrosis, which appears as a physical barrier to new vessel formation (discussed later), we cannot rule out the direct impact of CCN2/CTGF on the inhibition of angiogenesis. For example, in vitro studies show that CCN2/CTGF inhibits VEGF angiogenic action, which can be one of the mechanisms contributing to the low oxygen supply and hypoxic conditions in damaged or diseased skeletal muscle [67,73]. The importance of the role of CCN1/CYR-61 and CCN2/CTGF in the regulation of the stiffness vascular bed and its implications in physiological and pathological conditions has been recently reviewed [74].

## 3. CCN Proteins in Skeletal Muscle Fibrosis

The excessive accumulation of ECM proteins such as collagens, fibronectin, and proteoglycans, is denominated fibrosis [75]. Fibrosis severely affects different organs, replacing functional tissue, usually associated with chronic damage or disease and a robust inflammatory component [76,77,78,79]. In skeletal muscle, fibrosis can directly affect the structure and continuity of the endomysium, perimysium, and epimysium with tendons, interfering with muscle force transmission and changing the mechanical stimuli to myofibers and surrounding cells [75,80]. Fibrosis also modifies the concentration and availability of soluble factors and the interaction with receptors [75,81,82], and forms a physical barrier that extends on the damaged tissue, opposing physiological processes such as neovascularization, reinnervation, and proper regeneration [55,67,83,84].

The best-studied regulator of fibrosis in most tissues, including skeletal muscle, is transforming growth factor-β (TGF-β) [85,86,87,88]. Nevertheless, several CCN family members are now recognized as crucial modulators of fibrosis in diverse tissues [17]. Among the six members, CCN2/CTGF is the most studied CCN in the context of skeletal muscle fibrosis [22,27,89,90].

### 3.1. CCN2/CTGF Drives Skeletal Muscle Fibrosis

We have shown that CCN2/CTGF is overexpressed in fibrotic areas, characterized by massive deposition of ECM proteins, and in necrotic/regenerative cores in damaged muscle, acting as a profibrotic factor that upregulates ECM with detrimental effects in muscle function [89,91]. Consequently, when CCN2/CTGF is overexpressed in a normal and healthy muscle through adenoviral transduction, the muscle develops a fibrotic response, accumulating ECM proteins and showing reduced muscle function [92]. Interestingly, CCN2/CTGF expressed specifically in myofibers, and not in other neighbor cells, is upregulated by signals that appear during skeletal muscle damage and is responsible for the ECM remodeling and excessive accumulation in fibrotic muscle [22,68,90].

In skeletal muscle, CCN2/CTGF levels are elevated in diverse neuromuscular diseases, including muscular dystrophies of different etiology, muscle denervation, neurodegenerative diseases, and damage for muscle overuse [22,55,62,90,93,94,95,96,97,98,99,100,101]. In human dystrophic muscle, higher expression of CCN2/CTGF is associated with more significant fibrosis and severity of the disease [102]. CCN2/CTGF mRNA and protein levels increase very quickly after denervation of the skeletal muscle, followed by activation of other profibrotic pathways such as TGF-β signaling and ECM accumulation [100]. In a transgenic mouse model of ALS (tg hSOD1G93A), increased atrophy in post-symptomatic individuals is accompanied by elevated accumulation of ECM proteins such as fibronectin and collagens, increased TGF-β signaling, and overexpression of CCN2/CTGF [62,96,103].

Therapeutic approaches that can reduce skeletal muscle fibrosis are naturally considered since they can improve vascularization, reduce hypoxia, facilitate cell therapy and improve regeneration among other effects, finally allowing improvement of muscle function [55,83,84]. Because of the profibrotic role of CCN2/CTGF, and since its upregulation is a common pathological feature in most neuromuscular disorders, its downregulation or blockage has resulted in an exciting alternative for combating fibrosis and treating skeletal muscle diseases. Lower genetic load of *Ctgf* gene (*Ctgf^+/−^*) in dystrophic (*mdx*) mice leads to reduced muscle damage and apoptosis, improvement of tetanic muscle strength, and reduced fibrosis [55]. Furthermore, skeletal muscle dystrophy is ameliorated by systemic IP delivery of the specific antibody pamrevlumab (FG-3019, FibroGen Inc., San Francisco, CA, USA), which blocks CCN2/CTGF biological activity. Pamrevlumab treated *mdx* mice presented reduced fibrosis and improved muscle function compared with mice treated with control IgG [55]. First demonstrated in the *mdx* muscular dystrophy model, decreasing levels or activity of CCN2/CTGF can also decrease fibrosis and improve skeletal muscle performance in other models of different muscle pathologies. Thus, the fibrotic response resulting from muscle overuse injury in rats is reduced when CCN2/CTGF is blocked using pamrevlumab [101]. Denervation-associated fibrosis in mice is diminished in *Ctgf^+/−^* mice as well in mice treated with pamrevlumab [100]. In the transgenic hSODG93A mouse model of ALS, the systemic treatment with pamrevlumab delays the progression of locomotory symptoms and muscle wasting, enhances muscle performance, improves muscle architecture, and reduces fibrosis [62]. Remarkably, these studies have shown that in neuromuscular pathologies, the beneficial effects of blocking CCN2/CTGF activity can also improve or protect neuronal inputs. This effect on neurons is evidenced by decreased axon degeneration and preserved neuromuscular junction (NMJ) in the ALS model [62] and, in the rat model of muscle overuse, by amelioration of mononeuropathy, decreased neural fibrosis and less sensory decline [104], opening the possibility of using CCN2/CTGF as a target for neurodegenerative diseases [21,103]. Furthermore, the encouraging results of pamrevlumab in pre-clinical models lead to clinical trials for treating chronic skeletal muscle fibrosis in DMD patients. To date, pamrevlumab has achieved an open-label, single-arm Phase 2 clinical trial in non-ambulatory DMD patients (NCT02606136). Promising results from this trial indicate that the treatment helps to slow the decline and sometimes improves lung and heart function and skeletal muscle strength in patients with DMD [22,105]. The newly initiated phase 3 clinical trial LELANTOS (NCT04371666), is enrolling non-ambulatory DMD patients on a maintenance dose of corticosteroids, to evaluate pamrevlumab effectiveness.

### 3.2. Role of Other CCNs in Skeletal Muscle Fibrosis

Other CCNs different from CCN2/CTGF have also been involved in skeletal muscle fibrosis. CCN3/NOV is known to have an anti-fibrotic action in renal fibrosis models, downregulating CCN2/CTGF and decreasing the expression and accumulation of ECM proteins [106,107,108]. However, less is known about the anti-fibrotic role of CCN3/NOV in skeletal muscle. CCN3/NOV is downregulated by mechanical stress in fibroblasts, opposite to what occurs with CCN1 and CCN2 [41]. Similarly, in the rat model of muscle overuse, CCN2/CTGF is elevated while some isoforms of CCN3/NOV are reduced. Interestingly, treatment with pamrevlumab can restore levels of those CCN3/NOV isoforms [101], which the authors discuss as one of the mechanisms by which pamrevlumab is reducing fibrosis in the muscle overuse model. We previously reported that *Ccn3/Nov* mRNA levels are reduced in fibrotic skeletal muscle from a mouse model of ALS. However, treatment with pamrevlumab, which reduced fibrosis, did not significantly recover *Ccn3/Nov* mRNA levels [62], although CCN3/NOV protein isoforms were not analyzed. Furthermore, in vitro studies on rat primary palatal fibroblasts show that CCN3/NOV inhibits proliferation, induces apoptosis of fibroblasts, and decreases ECM proteins levels [109], which can be one of the mechanisms for the anti-fibrotic action of CCN3/NOV.

CCN5/WISP-2 is expressed in skeletal muscle tissue [110], but it is probably one of the less-studied CCN proteins in the context of skeletal muscle, together with CCN4/WISP-1 and CCN6/WISP-3. Nevertheless, we can speculate about its possible roles in skeletal muscle fibrosis based on its role in cardiac fibrosis. In cardiac muscle fibrosis associated with hypertension, CCN5/WISP-2 levels are reduced [111]. Comparable to CCN3/NOV in renal fibrosis, and opposite to fibrotic CCN2/CTGF, CCN5/WISP-2 is anti-fibrotic in the heart [112,113]. Its protective role occurs by inhibiting the AngII/TGF-β axis [111]. In vitro studies indicate that CCN5/WISP-2 can decrease the transdifferentiation to myofibroblasts and lead myofibroblasts to apoptosis [114], which can count as one mechanism for reducing cardiac fibrosis. Still, we cannot directly extrapolate this data because different mechanisms for CCN action have been observed in both types of muscles. For example, CCN2/CTGF is profibrotic in skeletal and cardiac muscle [22,115]. However, in the heart, fibroblast-derived CCN2/CTGF has fibrotic potential, while genetic manipulation of CCN2/CTGF in cardiomyocytes has no effect on the cardiac pathology, even when cardiomyocytes are the primary source of CCN2/CTGF [116,117]. On the other hand, in skeletal muscle, myofibers are the major source of CCN2/CTGF, and myofiber-derived CCN2/CTGF is upregulated in pathological conditions and carries fibrotic potential [68,90]. Therefore, further studies are needed to determine whether CCN5/WISP-2 has an anti-fibrotic function in skeletal muscle and what residing cell types might be involved.

## 4. Regulation of CCN2/CTGF and Fibrosis in Skeletal Muscle: Role of Vasoactive Peptides

The upregulation of CCN2/CTGF in fibrotic skeletal muscle responds to a conjunction of signaling pathways induced in conditions of damage or disease, which have been recently reviewed [22]. Between them, TGF-β has been widely studied. TGF-β transcriptionally upregulates several ECM proteins and CCN2/CTGF, which can increase TGF-β binding to its receptors, exacerbating TGF-β signaling in a positive feedback that promotes fibrosis [22,86,87,88,118]. The characteristic hypoxia of damaged tissue, with stabilization of hypoxia-inducible factor 1α (HIF-1α), is involved in establishing fibrosis in skeletal muscle by synergistically upregulate CCN2/CTGF in myotubes and myofibers when TGF-β is present, as in damaged skeletal muscle [22,67,68]. Also, the bioactive lipid lysophosphatidic acid (LPA), involved in inflammation and fibrosis in different tissues, including skeletal muscle [119], can induce CCN2/CTGF expression [50,52,120]. Some of the mechanisms that control the expression of CCN2/CTGF in response to LPA crosstalks with other signaling pathways such as TGF-β and JNK [50,52,120] and the hippo-YAP pathway [121]. The Yes-associated protein (YAP), the core effector of the Hippo pathway, and the TEAD transcription factors are critical regulators of muscle mass and mechanotransduction in skeletal muscle [122,123]. CCN2/CTGF and CCN1/CYR-61 are direct target genes of YAP/TEAD [124,125,126], and a novel regulatory pathway for angiogenesis in the retina has been described that comprises a loop between CCN2/CTGF and YAP [127]. Remarkably, YAP is upregulated in dystrophic *mdx* muscle, denervated muscle, and in hind-limbs from the SOD1G93A ALS model [122,128], then it is possible that YAP/TEAD can be contributing to CCN2/CTGF upregulation in fibrotic skeletal muscle, in a direct way and by crosstalking with TGF-β, LPA and hypoxia/vasculogenesis pathways. As a possible therapeutic target for kidney diseases, YAP inhibition using verteporfin effectively decreased renal fibrosis in mice, reducing TGF-β signaling and CCN2/CTGF levels [129]. Additional investigation must be performed to fully determine the contribution of YAP/TEAD on CCN2/CTGF modulation in fibrotic skeletal muscle.

Interestingly, proteins related to the cardiovascular system and blood pressure regulation have also been linked to the modulation of fibrosis. This section focuses on the regulation of CCN2/CTGF and skeletal muscle fibrosis by the renin-angiotensin and kallikrein-kinin systems of vasoactive peptides.

### 4.1. Vasoactive Peptides Modulate Fibrosis and CCN2/CTGF in Skeletal Muscle

**Figure 3:**
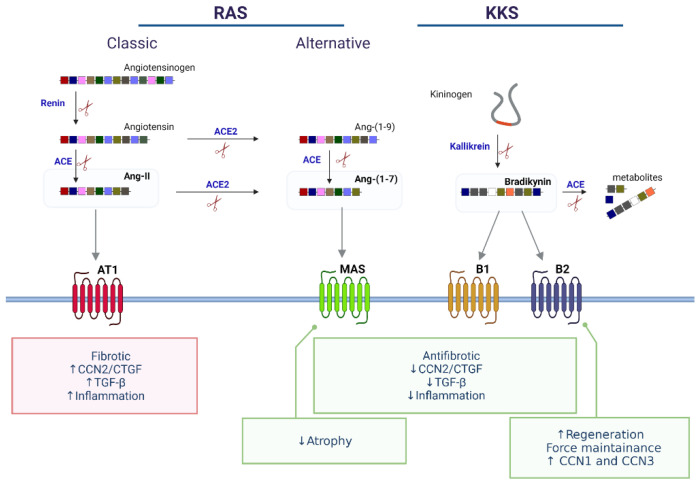
RAS and KKS in skeletal muscle. The subsequent enzymatic cleavage (shown as scissors) of Angiotensinogen and Kininogen, leads to the release of active peptides which activate specific receptors. The Renin-Angiotensin System (RAS) and the Kallikrein-Kinin System (KKS) of vasoactive peptides modulate CCN2/CTGF and possibly other CCNS, regulating skeletal muscle fibrosis. The activation of the canonical RAS promotes CCN2/CTGF, muscle fibrosis, and inflammation. On the other hand, alternative RAS and KKS protect skeletal muscle from damage and fibrosis. Figure created with BioRender.com (accessed on 14 May 2021).

Vasoactive peptides are biomolecules that regulate blood pressure, whose modulation is especially relevant for treating cardiovascular diseases. Nevertheless, the vasoactive peptides can also have local effects on several tissues [130,131]. In skeletal muscle, considerable evidence can be found in the literature showing that the pharmacological modulation of the renin-angiotensin system (RAS) and kallikrein kinin system (KKS) has beneficial effects on skeletal muscle in the context of different pathologies, including muscle atrophy, myopathies, muscular dystrophies, regeneration after trauma and sarcopenia, among others [132,133,134,135,136,137,138,139,140,141,142,143,144,145,146,147,148,149]. This section focuses on the RAS and KKS effects on skeletal muscle physiopathology that occur by the modulation of CCN proteins, with particular emphasis on the regulation of CCN2/CTGF and muscle fibrosis (summarized in Figure 3).

The RAS and KKS have precursor proteins that are cleaved by an enzyme, which releases the active peptides. This active peptide then binds to its G protein-coupled receptor (GPCR), activating signaling cascades that trigger different actions [130,131].

In the RAS, the enzyme renin cleaves angiotensinogen to produce angiotensin I (Ang-I, a ten amino acid peptide). Then, Ang-I can be further cleaved by the angiotensin-converting enzyme (ACE) to produce angiotensin II (Ang-II, an eight amino acid peptide). Ang-II binds to AT1 or AT2 receptors, which is the classic axis of the RAS. Alternatively, in the non-classical RAS axis, Ang-II can be cleaved by ACE2 to form angiotensin-(1–7) (Ang-(1-7), a seven amino acid peptide) that activates the Mas receptor, which has vasodilatory, anti-inflammatory, anti-fibrotic and anti-atrophic effects, all opposite to the classic axis [150] (Figure 3).

In the case of the KKS, kininogen is cleaved by kallikrein to form bradykinin (BK), which can bind B1 and B2 receptors, although it has more affinity to B2R [151] (Figure 3). B2Rs are constitutively expressed in several tissues, including skeletal muscle, while B1Rs are upregulated upon damage or inflammation. Frequently, the effects mediated by BK via B2 receptors are opposite to those of the classical axis of the RAS, being a potent vasorelaxant and antihypertensive agent [152]. RAS and KKS have different crosstalk points, where ACE is pivotal because it produces Ang-II and degrades BK. Consequently, the beneficial effects of ACE inhibitors (ACEi) stem from the reduction of Ang-II and the increase of BK [151,152]. In skeletal muscle, both RAS and KKS are involved in modulating muscle mass. Furthermore, in muscle dystrophies, the classic RAS has detrimental effects, and the non-classical RAS axis and KKS seem to be protective [147,150,153,154,155].

### 4.2. CCN2/CTGF Is Upregulated by the RAS Classical Axis in Skeletal Muscle and Promotes Fibrosis

The role of Ang-II on skeletal muscle fibrosis manifests when treating animal models of skeletal muscle dystrophy with losartan (an AT1 receptor blocker) or enalapril (an ACE inhibitor) [132,133]. These treatments reduce fibrosis, inflammation and improve muscle strength. Losartan reduces TGF-β expression levels and Smad-dependent signaling, an intracellular canonical signaling branch activated upon TGF-β binding to its receptor [133]. Since TGF-β upregulates CCN2/CTGF, and Ang-II induces CCN2/CTGF in models of hepatic, renal, and cardiac fibrosis [156,157,158,159], some studies were performed to evaluate the direct effect of losartan and enalapril treatment on CCN2/CTGF. In the *mdx* dystrophic mice, treatment with both losartan and enalapril reduced CCN2/CTGF levels in skeletal muscle. The CCN2/CTGF reduction correlated with a decrease in fibronectin and collagen, improved muscle histology and strength [148,149]. Similar effects were observed in a model of CCN2/CTGF overexpression since fibrosis was reduced and muscle force was increased by treatment with losartan and enalapril [149]. Only losartan treatment decreased TGF-β and Smad-dependent signaling in *mdx* mice, indicating that Smad activation (evidenced by Smad positive nuclei) is modulated by AT1 activity. Interestingly, in renal, vascular, and hepatic fibrosis models, Ang-II can induce Smad-dependent signaling through the AT1 receptor in a TGF-β-independent manner, increasing CCN2/CTGF levels. Therefore, this mechanism could also be occurring in skeletal muscle [156,158,160]. Additionally, in *mdx* mice, the treatment with both losartan and enalapril reduced CCN2/CTGF-induced ERK phosphorylation [148,149]. In skeletal muscle cells (C2C12 myoblasts), Ang-II induces NAD(P)H oxidase activation. In turn, ROS production led to increased CCN2/CTGF levels, and blocking both ROS and NAD(P)H oxidase reduced the increase in CCN2/CTGF. Further experiments confirmed that AT1 receptor and PKC activation are required for this effect [161]. Additional experiments in skeletal muscle cells showed that AT1 activation by Ang-II induces p38 and ERK phosphorylation, but only p38 activation is necessary for the fibrotic response induced by Ang-II. Ang-II-induced p38 phosphorylation is required to increase the levels of TGF-β, which is required for CCN2/CTGF upregulation since blocking the activity of the TGF-β receptor I or knocking down TGF-β by shRNA, reduced Ang-II induced CCN2/CTGF and fibrosis markers levels [162].

### 4.3. The Non-Classical RAS Axis and KKS Reduce CCN2/CTGF Levels in Models of Skeletal Muscle Fibrosis

The activation of the non-classical RAS axis is beneficial in skeletal muscle dystrophy [163]. In the *mdx* model of DMD, treatment with Ang-(1-7) plays a protective role reducing fibrosis, decreasing tissue damage, and improving muscle strength [146]. In the sarcoglycan-δ null mouse, a limb-girdle muscular dystrophy model, Ang-(1-7) treatment improved muscle function and reduced oxidative stress [164]. Further experiments indicate that the Ang-(1-7) effects are mediated by the Mas receptor signaling. Treatment of *mdx* mice with the Mas antagonist A-779, and the use of the knock out of the Mas receptor in *mdx* mice, result in worsening of the dystrophic phenotype, dramatically increasing damage, fibrosis, and Smad3 signaling, and reducing skeletal muscle strength [146]. We found that *Ccn2/Ctgf* mRNA levels decrease in *mdx* mice treated with Ang-(1-7) [146], and increase in the diaphragm of *mdx*KOMas mice (unpublished data). Interestingly, Ang-(1-7) can also reduce CCN2/CTGF levels in the muscle of a model of radiation-induced fibrosis [165]. Furthermore, TGF-β injection in skeletal muscle induces local damage and increases CCN2/CTGF levels. Treatment with Ang-(1-7) inhibits these effects [146].

KKS effect on skeletal muscle fibrosis was evaluated by inhibiting BK receptors in the *mdx* mice model. Two known inhibitors were used, des-Arg-Leu-BK (DALBK) and HOE-140, to inhibit B1R and B2R, respectively. These treatments caused an increment in CCN2/CTGF levels, worsening fibrosis and inflammation compared to non-treated *mdx* mice [147]. These results suggest that endogenous KKS actively protects skeletal muscle from a more severe dystrophic phenotype, and that one of the mechanisms is the reduction of CCN2/CTGF. When using antagonists of B1R and B2R, this endogenous protection is lost, and the fibrotic phenotype is aggravated. In the treated mice, CCN2/CTGF was increased not only in the endomysium and perimysium but also inside muscle fibers [147]. Interestingly, it is CCN2/CTGF derived from myofibers and not from fibroblasts, which had been previously demonstrated to play a role in modulating ECM and fibrosis in dystrophic muscle [90]. Although not evaluated in that study, the increment of CCN2/CTGF in myofibers when B1R and B2R were antagonized might be related to increased hypoxic signaling in the treated mice. CCN2/CTGF is synergistically upregulated in myofibers under hypoxia and the presence of TGF-β signaling, which is abundant in *mdx* skeletal muscle [67,68]. This hypothesis is supported by the fact that kinins have a role in vascularization. In a streptozotocin-induced diabetic mice model of hindlimb ischemia and reperfusion, the treatment with synthetic agonists of B1R and B2R leads to increased capillary density and muscle perfusion [166]. In the same diabetes model, the overexpression of human kallikrein promoted neovascularization, suppressed apoptosis, and upregulated endothelial nitric oxide synthase expression, protecting hindlimb from ischemic damage [167]. Another mechanism for hypoxia could be the impairment of the vascular vasodilatory response since in endothelial cells BK leads to a potent vasodilation response that can be mediated by nitric oxide (NO) through the activation of endothelial NO synthase (eNOS), prostacyclin (PGI_2_), and endothelial hyperpolarizing factor (EDHF) [168,169]. Interestingly, in the dog model of DMD, vascular endothelial dysfunction was reduced by bradykinin infusion, restoring the response to acetylcholine in the vascular bed, and effect that was dependent on NO, through the upregulation of eNOS and nNOS. nNOS is necessary for maintaining blood supply to myofibers in active muscles, and is misslocalized and downregulated in dystrophic muscle, which contribute to muscle pathology [170,171,172]. Then, inhibition of KKS can deteriorate the already hampered capillary network of the dystrophic muscle, decreasing oxygen supply, which can trigger a more extensive upregulation of CCN2/CTGF.

The inhibition of B2R with HOE-140 also caused a reduction in muscle force [147]. The fact that loss of muscle force occurs when B2R, but no B1R, is inhibited suggests a specific effect of BK acting through B2R that might be independent of CCN2/CTGF upregulation. Some evidence suggests that this differential effect on strength may be due to B2R influence on the differentiation of skeletal muscle progenitors. BK B2Rs are expressed in C2C12 myoblasts, and BK is secreted during differentiation. Treatment with HOE-140 to inhibit B2R increases myoblasts’ proliferation but reduces differentiation by downregulating desmin, myogenin, and myosin heavy chain expression [173]. Accordingly, after cardiotoxin injury, regeneration capacity is reduced in B2R knockout mice compared to wild type [173]. Given the role of CCNs on myoblast proliferation and differentiation discussed above, and because inhibition of B2R by infusion of HOE-140 increases CCN2/CTGF levels and decreases CCN1/CYR61 and CCN3/NOV expression [147], we cannot exclude a role of CCNs on the effects of B2R inhibition or absence on muscle regeneration. Further investigation is needed to evaluate the impact of KKS on muscle regeneration and its relationship with the CCN family.

### 4.4. Vasoactive Peptides and Other CCN Proteins

The influence of vasoactive peptides on other CCNs different from CCN2/CTGF, has been poorly explored in skeletal muscle. However, what is known in cardiac muscle offers clues and suggests some hypotheses. CCN1/CYR61 is often studied along with CCN2/CTGF in mice models of cardiac infarction, where both CCNs are induced 12 o 14 weeks after induction of myocardial infarction. Ang-II modulates its expression since treatment with telmisartan (an AT1 receptor blocker) reduces CCN1/CYR61 levels [174,175]. Interestingly, in these models, telmisartan reduced CCN2/CTGF levels only if the treatment was started at the beginning of infarction [174]. When the treatments began 36–48 h after infarction, only CCN1/CYR61 was reduced [175], highlighting the importance of understanding the time frames for effective therapeutic intervention. Other studies in models for chronic cardiac failure indicate that Ang-II mediates cardiac hypertrophy through the induction of CCN4/WISP-1 [176,177]. Ang-II increases CCN4/WISP-1 mRNA and protein levels through a mechanism involving activation of the AT1 receptor, via NOX2-induced superoxide/Akt/GSK3β/β-catenin/TCF/LEF signaling and via superoxide-mediated p38 MAPK- and ERK1/2-dependent CREB activation [177]. Ang-II serum and cardiac levels, which are increased in a model of spontaneous hypertensive rats (SHR), are reduced by treatment with enalapril with a concomitant increment in CCN5/WISP-2. Furthermore, there is a progressive increase in Ang-II and CCN2/CTGF levels that positively correlates with the degree of hypertension (blood pressure values) in patients. In contrast, a reduction in CCN5/WISP-1 levels is observed. In cardiac fibroblasts, Ang-II reduces CCN5/WISP-2, and siRNA against *Ccn5/Wisp-2* increases the basal levels of the fibrotic markers TGF-β, collagen, and α-SMA [111]. Interestingly, transgenic mice expressing CCN5/WISP-2 were protected against cardiac hypertrophy and fibrosis due to overload pressure, with reduced Smad2/3 and Smad4 levels. Also, phycoerythrin- or CCN2-induced hypertrophy was reduced in cardiomyocytes overexpressing CCN5/WISP-2 [112]. These data indicate that CCN5/WISP-2, which is downregulated by Ang-II, functions as an anti-fibrotic molecule in the heart. Further investigation is needed to determine whether the same anti-fibrotic role of CCN5/WISP-2 is present in the skeletal muscle. If this is the case, the upregulation of CCN5/WISP-2 can be another mechanism to reduce skeletal muscle fibrosis when AT1 or ACE are inhibited, besides the downregulation of CCN2/CTGF.

## 5. Summary and Concluding Remarks

The CCN family of proteins regulates cell signaling pathways in skeletal muscle, which functions in physiological processes to maintain muscle health. Alterations in CCNs levels and function contribute to the development of pathological conditions. Consequences in the skeletal muscle include abnormalities in skeletal muscle regeneration and muscle mass regulation, decreased vascularization and oxygen availability, modulation of ECM, and fibrosis establishment. One of the most studied CCN in skeletal muscle pathology is CCN2/CTGF, evidenced by gain and loss of function approaches in pre-clinical models. Furthermore, the understanding of CCN2/CTGF function and its role in fibrosis in different organs, including skeletal muscle, has led to therapeutic strategies using antibodies to block CCN2/CTGF activity. Currently, clinical trials have used pamrevlumab to evaluate the treatment of fibrosis associated with DMD. Moreover, successful pre-clinical studies in other neuromuscular diseases will hopefully extend the use of therapeutic antibodies to other neuromuscular diseases where fibrosis is a prominent characteristic.

Although knowledge exists for the mechanisms and possible cell sources of CCN2/CTGF (muscle fibers) that specifically participate in the fibrotic process, less information is available for other CCNs in different muscle pathology aspects. Therefore, efforts must be made in the deep understanding of the role and mechanisms of other CCNs in skeletal muscle pathology, particularly in the context of muscle wasting, which is also a characteristic of many diseases such as sarcopenia, cachexia, muscle denervation, and disuse.

There is good evidence about the beneficial effects of modulating vasoactive systems, RAS and KKS, to reduce muscle pathology, making them good therapeutic alternatives. Furthermore, there is growing evidence that one of the mechanisms for these effects, especially for reducing fibrosis, is through the modulation of CCN2/CTGF. More research is necessary to understand better the role of RAS and KKS on other CCNs in the context of skeletal muscle disease.
